# Supplementary material for: Current definitions of advanced multimorbidity: a protocol for a scoping review
Source: BMJ Open. 2023 Nov 30;13(11):e076903. doi: 10.1136/bmjopen-2023-076903 (PMC10689385; doi:10.1136/bmjopen-2023-076903)
Supplement: Supplementary data [file bmjopen-2023-076903supp001.pdf]

Medline and Embase

1. Multimorbidity/
2. (multimorbid* or multi-morbid* or polymorbid* or poly-morbid* or multicondition* or multi-condition* or ((multiple or coexist* or co-exist* or concurrent or co-occur*) adj (comorbid* or co-morbid* or "long term" or long-term or chronic or disease* or illness* or diagnos* or morbid* or condition*))).ti,ab
3. 1 or 2
4. Palliative Care/
5. Terminal Care/
6. (palliat* or terminal or "end of life" or end-of-life or dying or died or "last year" or "final year" or ((final or last) adj1 month*)).ti,ab.
7. 4 or 5 or 6
8. 3 and 7
9. (("end stage" or end-stage or "advanc*" or "complex") adj2 (multimorbid* or multi-morbid* or polymorbid* or poly-morbid* or multicondition* or multi-condition* or ((multiple or coexist* or co-exist* or concurrent or co-occur*) adj (comorbid* or co-morbid* or "long term" or long-term or chronic or disease* or illness* or diagnos* or morbid* or condition*)))).ti,ab
10. 8 or 9

This search strategy has been chosen as described in our Methods section to capture key papers around themes of ‘multimorbidity’, ‘end of life’ and ‘palliative care’.

Steps 1-3 capture multimorbidity

Steps 4-7 capture end of life care and palliative care

Step 8 then combines the above phrases to focus these above points on when multimorbidity is described at the end of life or in a palliative care context

Step 9 captures papers which specifically mention advanced (or other synonymous phrases) multimorbidity

Steps 8 and 10 is then our final included papers in the search strategy
