# Supplementary material for: Current definitions of advanced multimorbidity: a protocol for a scoping review
Source: BMJ Open. 2023 Nov 30;13(11):e076903. doi: 10.1136/bmjopen-2023-076903 (PMC10689385; doi:10.1136/bmjopen-2023-076903)
Supplement: Author's manuscript [file bmjopen-2023-076903.draft_revisions.pdf]

BMJ Open

BMJ Open is committed to open peer review. As part of this commitment we make the peer review history of every article we publish publicly available.

When an article is published we post the peer reviewers' comments and the authors' responses online. We also post the versions of the paper that were used during peer review. These are the versions that the peer review comments apply to.

The versions of the paper that follow are the versions that were submitted during the peer review process. They are not the versions of record or the final published versions. They should not be cited or distributed as the published version of this manuscript.

BMJ Open is an open access journal and the full, final, typeset and author-corrected version of record of the manuscript is available on our site with no access controls, subscription charges or pay-per-view fees (http://bmjopen.bmj.com).

If you have any questions on BMJ Open's open peer review process please email info.bmjopen@bmj.com

BMJ Open

Current definitions of advanced multimorbidity: a scoping review.

Journal:	*BMJ Open*
Manuscript ID	bmjopen-2023-076903
Article Type:	Protocol
Date Submitted by the Author:	20-Jun-2023
Complete List of Authors:	Bowers, Sarah; University of St Andrews School of Medicine<br>McCheyne, Lewis; NHS Tayside<br>Wilson, Darcy; University of St Andrews School of Medicine<br>Mills, Sarah; University of St Andrews School of Medicine; NHS Fife<br>Agrawal, Utkarsh; University of Oxford Nuffield Department of Primary Care Health Sciences<br>Williams, Linda; University of Edinburgh, Usher Institute<br>Quirk, Frances; University of St Andrews School of Medicine; NHS Fife<br>Bowden, Jo; University of St Andrews School of Medicine; NHS Fife
Keywords:	PALLIATIVE CARE, Decision Making, INTERNAL MEDICINE, Life Change Events, Adult palliative care < PALLIATIVE CARE, Patient-Centered Care

SCHOLARONE™
Manuscripts

Title

Current definitions of advanced multimorbidity: a scoping review.

Dr Sarah P Bowers

Corresponding author

School of Medicine, University of St Andrews, St Andrews, UK

School of Medicine, North Haugh Street, St Andrews, KY16 9TF

01334 463599

sb461@st-andrews.ac.uk

Dr Lewis McCheyne

NHS Tayside, Dundee, UK

lewis.mccheyne@nhs.scot

Dr Darcy Wilson

School of Medicine, University of St Andrews, St Andrews, UK

dsw4@st-andrews.ac.uk

Dr Sarah E E Mills

School of Medicine, University of St Andrews, St Andrews, UK

NHS Fife, Fife, UK

seem1@st-andrews.ac.uk

Dr Utkarsh Agrawal

Nuffield Department of Primary Care Health Sciences, University of Oxford, Oxford, UK

utkarsh.agrawal@phc.ox.ac.uk

Dr Linda Williams

Edinburgh Clinical Trials Unit, Usher Institute, The University of Edinburgh, Edinburgh, UK

linda.williams@ed.ac.uk

For peer review only

Professor Frances Quirk

School of Medicine, University of St Andrews, St Andrews, UK

NHS Fife, Fife, UK

frances.quirk@nhs.scot

Dr Jo Bowden

School of Medicine, University of St Andrews, St Andrews, UK

NHS Fife, Fife, UK

joanna.bowden@nhs.scot

For peer review only

Word Count: 2255

Keywords:

Multimorbidity

Palliative Care

Terminal Care

Review

Article Summary

Strengths & Limitations

- This is the first scoping review to identify and map out how people with multimorbidity who are approaching the end of their life are identified in research, policy, and practice
- This protocol has been developed robustly using international guidelines and will be reported using the Preferred Reporting Items for Systematic Review and Meta-Analyses extension for Scoping Reviews (PRISMA-ScR) guidelines
- Utilising content analysis will allow for definitions from various bodies of work to be described narratively and aid future researchers, policy makers and clinicians
- A project Patient Advisory Group has been established who participated in conceptualisation of this scoping review
- This work is limited to English language but there is no date limit utilised

For peer review only

Abstract (word count = 286)

Introduction

People living with and dying from multimorbidity are increasing in number, and ensuring quality care for this population is acknowledged to be one of the major challenges currently facing healthcare providers. People with multimorbidity have been shown to have a high burden of palliative and end of life care needs, though they do not always access specialist palliative care services. A key reason for this is that they are often not identified as being in the last stages of their life by current healthcare providers and systems.

This scoping review aims to describe how existing research and policies define advanced multimorbidity and to present these findings in a way that highlights commonalities between definitions that could be operationalised into practice.

Methods and analysis

Scoping review methodology, based on Arksey and O'Malley's framework, will be undertaken and presented using the Preferred Reporting Items for Systematic Review and Meta-Analyses extension for Scoping reviews (PRISMA-ScR). Search terms have been generated using the key themes of 'multimorbidity', 'end of life' and 'palliative care'. Peer-reviewed research will be obtained through systematic searching of Medline, EMBASE, CINAHL, Scopus and PsychINFO. Grey literature will be searched in a systematic manner. After screening studies for eligibility, included studies will be described in terms of setting and characteristics as well as using inductive content analysis to highlight the commonalities in definitions.

Ethics and dissemination

Ethical approval is not required for this scoping review. The findings of the scoping review will be used internally as part of SPB's PhD thesis at the University of St Andrews through the Multimorbidity Doctoral Training Programme for Health Professionals, which is supported by the Wellcome Trust (223499/Z/21/Z) and published in an open access, peer reviewed journal for wider dissemination.

For peer review only

Introduction

Multimorbidity, defined as the presence of two or more physical or mental health conditions, is one of the main challenges facing modern healthcare systems.(1) The prevalence of multimorbidity is increasing and is particularly high in older adults, those living with socioeconomic deprivation and those with mental health disorders.(2) People living with multimorbidity are known to have poorer quality of life, increased healthcare utilisation and expenditure and shorter life expectancy.(3, 4, 5, 6) There has been criticism that most clinical guidelines and healthcare systems are designed to focus only on discrete single-disease illnesses, with little account for the interplay that having concurrent illnesses has on both disease and patient-related factors.(1, 7, 8) The National Institute for Care and Excellence (NICE) subsequently published a guideline on managing multimorbidity with an aim to reduce treatment burden and unplanned care through providing individualised management plans and patient-centred care.(9) This guidance acknowledges that further research is needed to identify people with multimorbidity who have a reduced life expectancy; however the focus of this is to then aid decision making about stopping or starting preventive medication rather than necessarily undertaking holistic assessment of those near the end of their life.

People with multimorbidity face the prospect that any or all of their conditions may be incurable, either due to the nature of the individual conditions or due to the interplay of the different conditions.(10, 11, 12) For people with incurable single disease conditions, identification of potential unmet palliative and end of life care needs is supported by various guidelines which advocate for the proactive adoption of a palliative care approach and, where needed, early referral to palliative care services.(13, 14) Palliative care is defined by the World Health Organisation (WHO) as: "an approach that improves the quality of life of patients (adults and children) and their families who are facing problems associated with life-threatening illness. It prevents and relieves suffering through the early identification, correct assessment and treatment of pain and other problems, whether physical, psychosocial or spiritual."(15) It can be delivered by both generalists (e.g. general practitioners, secondary care practitioners) or specialists within the field of palliative care.(16) Involvement with palliative care specialists, particularly at an early stage, has been shown to have benefits on patient quality of life and in certain circumstances improve survival.(17, 18) As per the WHO definition, people with multiple long-term conditions have been shown to have a variety of palliative care needs, and there is some evidence these are comparable to those with incurable cancer.(19, 20) Prediction modelling has also demonstrated that people with multiple long-term conditions are going to be the highest users of palliative care services by 2040.(21) Despite acknowledging the high level of need, people with multimorbidity continue to be under-referred to palliative care services, and/or referred too late.(20, 22)

Difficulty in recognising the palliative care needs of people with multimorbidity may arise from challenges in identifying when they are approaching the end of life, and pursuit of disease-modifying treatments may no longer be beneficial.(23) This so called 'advanced illness' has previously been defined in the literature as 'late-stage chronic illness when one or more conditions become serious enough that general health and function decline and treatments begin to lose their impact – a state that progresses to end of life'.(24) It is recognised that it can be a challenge to identify the end of life period for people with multimorbidity due to the unpredictable disease trajectory they often have, which may in part be due to the heterogeneity of multimorbidity itself.(25) Providing the opportunity for identification of an imminence of the end of life would allow patients with multimorbidity and their loved ones the opportunity to express preferences for care and access formalised social, health and bereavement support.(26, 27) However at present, there is no accepted definition or guidance for when in the multimorbidity illness journey a palliative care

For peer review only

approach may be beneficial. In other words, the lack of an agreed working definition of 'advanced multimorbidity' risks people with multimorbidity who are nearing the end of life, and their caregivers, missing out on important care and support.

Aim & Objectives

In order to comprehensively detail how advanced multimorbidity might be defined for use in future research, practice and policy, this scoping review aims to identify and present the available evidence on how people with multimorbidity are currently included in research, policy and clinical practice. This review will particularly focus on when the goals of care of those with multimorbidity is moved from solely aiming to reverse or treat chronic conditions, and instead focuses on alleviating suffering and addressing multidimensional needs – in line with a 'palliative care approach'.(15) Specifically, the objectives within this scoping review are to:

- Describe how states of advanced multimorbidity are operationalised within published research and end of life care policies
- Summarise the characteristics and methodologies of the studies examining or including advanced multimorbidity
- Present the limitations and identify research gaps within this pre-established knowledge.

Methods

Scoping review methodology was selected as this particular review type has a role in conceptual mapping in healthcare, particularly in areas without a universally agreed definition.(28) This scoping review will adhere to the five-step Arksey and O'Malley framework for scoping reviews, with reference to both the updates to this from Levac et al and the Joanna Briggs Institute (JBI) guidance for conducting scoping reviews.(29, 30, 31) This scoping review was reported in accordance with the Preferred Reporting Items for Systematic Review and Meta-Analyses extension for Scoping Reviews (PRISMA-ScR).(32)

Stage 1: Identifying the Research Question

The main objective of this study is to identify how advanced multimorbidity is defined in research, guidelines, policies and clinical practice. We have conceptualised that, as per other single-disease conditions, advanced multimorbidity would encompass such patients who have multimorbidity that are in a 'terminal phase' of that illness experience where it is expected that they are likely to die and therefore focuses of care should, reasonably, shift towards a comfort approach. This is in keeping with standard accepted definitions of other life-limiting conditions and reviews on language around what encompasses an advanced illness (24, 33, 34, 35, 36) Understanding the definitions of advanced multimorbidity that are already used in research and clinical practice will allow for informed research exploring the needs of this group of patients and how they are identified for both research, clinical care and policy.

Stage 2: Identifying Relevant Studies

SPB will identify relevant studies through searches of peer-reviewed and grey literature. Search terms relating to multimorbidity, advanced illness and palliative care were developed with expertise from the University of St Andrews library service to build our search strategy, see supplementary file 1. Patients involved in health research on the impact of multimorbidity, alongside our own public

advisors for this work, have denounced the term multimorbidity as having negative terms, with preferences being made for 'multiple long-term conditions', with literature often using the terms interchangeably and therefore we have integrated multiple long-term conditions into our search terms.(37) The databases Medline, EMBASE, CINAHL, Scopus and PsychINFO will be searched for published journal articles.

There will also be a search of grey literature within the United Kingdom to identify both unpublished work and clinical guidelines and policies. Following the guidance set out by Godin et al(38), our grey literature search will comprise of a four-step approach of: searching a grey literature database – National Grey Literature Collection, supported by Health Education England(39); a contained Google search; browsing targeted websites – related to end of life care in the UK; and a discussion amongst the clinical members of the research team (SPB, LM, DW, SM, JB) who have experience in generalist and specialist palliative care in various capacities to act as content experts and identify other items for possible inclusion in the review.

The references of all included reports or articles will be searched for additional studies. There will be no date limit on our searches though we will limit to English language.

Stage 3: Study Selection

Once all searches are complete these will be combined and deduplicated using EndNote X7. Using the inclusion and exclusion criteria (Table 1), developed using the JBI Population-Concept-Context model(29), titles and abstracts will be screened by two independent reviewers (SPB, LM and DW). We have particularly chosen to exclude studies on index conditions and comorbidity as although some argue that comorbidity and multimorbidity are interchangeable, the former is disease-focussed whereas the use of multimorbidity allows for delivery of more holistic, patient-centred care.(40) The full texts of selected citations will then be reviewed by two independent reviewers (SPB, LM and DW) to allow for approval for final study selection. Any discrepancies that may arise from each reviewer will be relayed back to a third reviewer and discussions will be held to reach consensus. Each stage of this study selection will be reported using the PRISMA-ScR Flow Chart.(32)

**Inclusion**	**Exclusion**
Population	
Adults (>18) with multimorbidity (≥2 health conditions) or their carers or healthcare providers	Studies focusing on one index condition<br>Studies focusing on comorbidity
Concept	
Definitions of multimorbidity in a terminal phase of the illness experience	Studies related solely to prognosis<br>No clear definition for advanced multimorbidity in the study/report
Context	
All settings and countries<br>Original research articles and review articles	Unpublished research<br>Not English language

Table 1 – Inclusion & Exclusion Criteria

Stage 4: Charting the Data

Data will be extracted by two independent reviewers to an Excel spreadsheet via the University of St Andrews OneDrive system to allow all researchers access – known as 'data charting' in scoping

For peer review only

reviews.(29) Charted data will relate to study design, study population, definition of multimorbidity and reported outcomes, summarised in Table 2. This will be an iterative approach with opportunity for updating the data-charting form as informed by the literature at regular research team meetings. Given that scoping reviews aim to assess the breadth of the evidence base, formal quality assessment is not needed.(29)

**Study Citation Information**	Author<br>Title<br>Journal<br>Year of publication
**Study Objective**	Aim and/or objectives
**Study Methods**	Type of study
**Study Characteristics**	Country<br>Healthcare setting (if applicable)<br>Participants (number, gender, diagnoses)<br>Inclusion/exclusion criteria
**Extracted results**	Definition for advanced multimorbidity<br>Findings related to aim of study<br>Other relevant information

Table 2 – Data Charting

Stage 5: Collating, summarising and reporting the results

Once we have identified all available definitions in the literature, we will analyse the data, report the results and apply meaning to the results. We will firstly summarise the characteristics of the included work descriptively by listing the overall number of studies, distribution of the work, study populations, and countries. Using content analysis, we will explore the different definitions that are found. Content analysis allows for analysis of both quantitative and qualitative research and policies to describe phenomena to make replicable and valid inferences about the data and provide a practical guide to action. It has been successfully utilised in other scoping reviews to assimilate data on definitions.(41, 42, 43) Given there is no formally accepted standard definition of advanced multimorbidity we will undertake our content analysis inductively which involves three main phases:

- Preparation phase: wherein the unit of analysis (in this case the definition) is selected from the data using key words and phrases and a description of the context in which this was obtained
- Organisation phase: open coding is then used to collate broad categories to describe the definitions which are then organised
- Abstraction phase: this formulates a general description of the research topic through the categories above and will then be described narratively.(44)

We will invite our project's public advisors to add their perspective to our results, particularly at the abstraction phase to ensure these are meaningful and relevant to people with lived experience.

Ethical Approval and Dissemination

This scoping review will not require ethical approval.

The findings of this scoping review will be shared through professional networks, conferences and published open access in a peer-reviewed journal.

For peer review only

Patient and Public Involvement

The concept and aim of this scoping review has been reviewed by our public advisors, who are members of the Fife Community Advisory Council providing patient and public involvement to the University of St Andrews. The group felt that identifying the dying phase of an individual's life was important, though also emphasised that understanding of the individual at the core of this was of most important. This lead us to utilise more holistic, patient-centred themes to our search terms (including multiple long-term conditions and the decision to focus on multimorbidity and not comorbidities). The group also feel that uncoordinated care was a challenge for people at the end of life and it is hoped this scoping review will provide operational suggestions of ways to identify people to then provide holistic, coordinated services in the future.

Discussion

This scoping review aims to comprehensively analyse the currently used definitions to identify people with multimorbidity who may soon be approaching the end of life. Identifying patterns to these definitions, alongside the input of our expert focus groups, will allow for open discussion and consensus to be reached on which are the important issues to consider when identifying advanced multimorbidity. This will primarily allow for earlier advanced care planning and opportunities for specialist care for patients with multimorbidity as well as ensuring more consistent research is undertaken and impact on national and international policy development.

Funding Statement

This review will contribute to the primary author's (SPB) PhD Thesis. SPB is a fellow on the Multimorbidity Doctoral Training Programme for Health Professionals, which is supported by the Wellcome Trust (223499/Z/21/Z), and supervised by SEEM, UA, LW, JB and FQ. As this research was funded in whole, or in part, by the Wellcome Trust (223499/Z/21/Z), for the purpose of open access, the author has applied a CC BY public copyright licence to any Author Accepted Manuscript version arising from this submission.

Acknowledgements

We are extremely grateful to Vicki Cormie, Senior Librarian at University of St Andrews, for her support and guidance in the development of the search strategy and to our public advisory team who have provided their expert lived opinion to the development of this scoping review. Particular thanks also to Rosie Dunn for her guidance on using content analysis within scoping reviews.

Author contributions

SPB developed the concept of the scoping review, research questions, public advisory group discussions and drafted the manuscript. SEEM and UA assisted with development of the search strategy. SPB, LM, DW, SEEM and JB provided content guidance through their clinical expertise. SPB

For peer review only

drafted the manuscript with LM, DW and JB critically reviewing drafts of this. All authors approved the final submitted manuscript and agree to be accountable for all aspects of this protocol.

For peer review only

References

1. Whitty CJM, MacEwen C, Goddard A, Alderson D, Marshall M, Calderwood C, et al. Rising to the challenge of multimorbidity. Bmj. 2020;368:l6964.
2. Barnett K, Mercer SW, Norbury M, Watt G, Wyke S, Guthrie B. Epidemiology of multimorbidity and implications for health care, research, and medical education: a cross-sectional study. Lancet. 2012;380(9836):37-43.
3. Lawson KD, Mercer SW, Wyke S, Grieve E, Guthrie B, Watt GC, et al. Double trouble: the impact of multimorbidity and deprivation on preference-weighted health related quality of life a cross sectional analysis of the Scottish Health Survey. Int J Equity Health. 2013;12:67.
4. Soley-Bori M, Ashworth M, Bisquera A, Dodhia H, Lynch R, Wang Y, et al. Impact of multimorbidity on healthcare costs and utilisation: a systematic review of the UK literature. Br J Gen Pract. 2021;71(702):e39-e46.
5. Johnston MC, Black C, Mercer SW, Prescott GJ, Crilly MA. Prevalence of secondary care multimorbidity in mid-life and its association with premature mortality in a large longitudinal cohort study. BMJ Open. 2020;10(5):e033622.
6. He K, Zhang W, Hu X, Zhao H, Guo B, Shi Z, et al. Relationship between multimorbidity, disease cluster and all-cause mortality among older adults: a retrospective cohort analysis. BMC Public Health. 2021;21(1):1080.
7. Banerjee S. Multimorbidity--older adults need health care that can count past one. Lancet. 2015;385(9968):587-9.
8. Guthrie B, Payne K, Alderson P, McMurdo ME, Mercer SW. Adapting clinical guidelines to take account of multimorbidity. Bmj. 2012;345:e6341.
9. NICE. Multimorbidity: clinical assessment and management 2016 [Available from: https://www.nice.org.uk/guidance/ng56.
10. Yardley S, Cottrell E, Rees E, Protheroe J. Modelling successful primary care for multimorbidity: a realist synthesis of successes and failures in concurrent learning and healthcare delivery. BMC Fam Pract. 2015;16:23.
11. Navickas R, Petric VK, Feigl AB, Seychell M. Multimorbidity: What do we know? What should we do? J Comorb. 2016;6(1):4-11.
12. Brijoux T, Woopen C, Zank S. Multimorbidity in old age and its impact on life results. Z Gerontol Geriatr. 2021;54(Suppl 2):108-13.
13. Ferrell BR, Temel JS, Temin S, Alesi ER, Balboni TA, Basch EM, et al. Integration of Palliative Care Into Standard Oncology Care: American Society of Clinical Oncology Clinical Practice Guideline Update. J Clin Oncol. 2017;35(1):96-112.
14. Jordan K, Aapro M, Kaasa S, Ripamonti CI, Scotté F, Strasser F, et al. European Society for Medical Oncology (ESMO) position paper on supportive and palliative care. Ann Oncol. 2018;29(1):36-43.
15. WorldHealthOrganisation. Palliative care 2020 [Available from: https://www.who.int/news-room/fact-sheets/detail/palliative-care.
16. NICE. Palliative care - general issues: What is palliative care? 2022 [Available from: https://cks.nice.org.uk/topics/palliative-care-general-issues/background-information/definition/.
17. Gaertner J, Siemens W, Meerpohl JJ, Antes G, Meffert C, Xander C, et al. Effect of specialist palliative care services on quality of life in adults with advanced incurable illness in hospital, hospice, or community settings: systematic review and meta-analysis. Bmj. 2017;357:j2925.
18. Temel JS, Greer JA, Muzikansky A, Gallagher ER, Admane S, Jackson VA, et al. Early palliative care for patients with metastatic non-small-cell lung cancer. N Engl J Med. 2010;363(8):733-42.
19. Nicholson C, Davies JM, George R, Smith B, Pace V, Harris L, et al. What are the main palliative care symptoms and concerns of older people with multimorbidity?-a comparative cross-sectional study using routinely collected Phase of Illness, Australia-modified Karnofsky Performance Status and Integrated Palliative Care Outcome Scale data. Ann. 2018;7(Suppl 3):S164-S75.

For peer review only

20. Llop-Medina L, Fu Y, Garces-Ferrer J, Donate-Martinez A. Palliative Care in Older People with Multimorbidities: A Scoping Review on the Palliative Care Needs of Patients, Carers, and Health Professionals. Int J Environ Res Public Health. 2022;19(6):08.
21. Finucane AM, Bone AE, Etkind S, Carr D, Meade R, Munoz-Arroyo R, et al. How many people will need palliative care in Scotland by 2040? A mixed-method study of projected palliative care need and recommendations for service delivery. BMJ Open. 2021;11(2):e041317.
22. Carduff E, Johnston S, Winstanley C, Morrish J, Murray SA, Spiller J, et al. What does 'complex' mean in palliative care? Triangulating qualitative findings from 3 settings. BMC Palliat Care. 2018;17(1):12.
23. Tobin J, Rogers A, Winterburn I, Tullie S, Kalyanasundaram A, Kuhn I, et al. Hospice care access inequalities: a systematic review and narrative synthesis. BMJ Supportive & Palliative Care. 2022;12(2):142-51.
24. Stuart B, Mahler E, Koomson P. A Large-Scale Advanced Illness Intervention Informs Medicare's New Serious Illness Payment Model. Health Aff (Millwood). 2019;38(6):950-6.
25. Teraoka E, Kunisawa S, Imanaka Y. Trajectories of end-of-life medical and long-term care expenditures for older adults in Japan: retrospective longitudinal study using a large-scale linked database of medical and long-term care claims. BMC geriatr. 2021;21(1):403.
26. NICE. Quality standard [QS13]. End of life care for adults: National Institute for Health and Care Excellence; [updated 02/09/2021. Available from: https://www.nice.org.uk/guidance/qs13#:~:text=This%20quality%20standard%20covers%20care,with%20life%2Dthreatening%20acute%20conditions.
27. Kennedy C, Brooks-Young P, Brunton Gray C, Larkin P, Connolly M, Wilde-Larsson B, et al. Diagnosing dying: an integrative literature review. BMJ Supportive & Palliative Care. 2014;4(3):263-70.
28. Anderson S, Allen P, Peckham S, Goodwin N. Asking the right questions: scoping studies in the commissioning of research on the organisation and delivery of health services. Health Res Policy Syst. 2008;6:7.
29. Peters MD, Godfrey CM, Khalil H, McInerney P, Parker D, Soares CB. Guidance for conducting systematic scoping reviews. Int J Evid Based Healthc. 2015;13(3):141-6.
30. Arksey H, O'Malley L. Scoping studies: towards a methodological framework. International Journal of Social Research Methodology. 2005;8(1):19-32.
31. Levac D, Colquhoun H, O'Brien KK. Scoping studies: advancing the methodology. Implement Sci. 2010;5:69.
32. Tricco AC, Lillie E, Zarin W, O'Brien KK, Colquhoun H, Levac D, et al. PRISMA Extension for Scoping Reviews (PRISMA-ScR): Checklist and Explanation. Ann Intern Med. 2018;169(7):467-73.
33. Hudson RB, Lindberg BW. Advanced Illness Care: Issues and Options. Public Policy & Aging Report. 2014;24(3):79-80.
34. Bruera E, Yennurajalingam S. Palliative care in advanced cancer patients: how and when? Oncologist. 2012;17(2):267-73.
35. Traue DC, Ross JR. Palliative care in non-malignant diseases. J R Soc Med. 2005;98(11):503-6.
36. Hui D, Nooruddin Z, Didwaniya N, Dev R, De La Cruz M, Kim SH, et al. Concepts and definitions for "actively dying," "end of life," "terminally ill," "terminal care," and "transition of care": a systematic review. J Pain Symptom Manage. 2014;47(1):77-89.
37. Chew-Graham C, O'Toole L, Taylor J, Salisbury C. 'Multimorbidity': an acceptable term for patients or time for a rebrand? Br J Gen Pract. 2019;69(685):372-3.
38. Godin K, Stapleton J, Kirkpatrick SI, Hanning RM, Leatherdale ST. Applying systematic review search methods to the grey literature: a case study examining guidelines for school-based breakfast programs in Canada. Syst. 2015;4(1):138.
39. Health Education England. National Grey Literature Collection 2023 [Available from: https://allcatsrgrey.org.uk/wp/.

For peer review only

40. Harrison C, Fortin M, van den Akker M, Mair F, Calderon-Larranaga A, Boland F, et al. Comorbidity versus multimorbidity: Why it matters. J Multimorb Comorb. 2021;11:2633556521993993.
41. Dunn R, Clayton E, Wolverson E, Hilton A. Conceptualising comorbidity and multimorbidity in dementia: A scoping review and syndemic framework. J Multimorb Comorb. 2022;12:26335565221128432.
42. Kwok E, Bootsma J, Cahill P, Rosenbaum P. A scoping review of qualitative studies on parents' perspectives on speech, language, and communication interventions. Disability and Rehabilitation. 2022;44(25):8084-93.
43. Churruca K, Pomare C, Ellis LA, Long JC, Braithwaite J. The influence of complexity: a bibliometric analysis of complexity science in healthcare. BMJ Open. 2019;9(3):e027308.
44. Elo S, Kyngäs H. The qualitative content analysis process. J Adv Nurs. 2008;62(1):107-15.

For peer review only

Medline and Embase

1. Multimorbidity/
2. (multimorbid* or multi-morbid* or polymorbid* or poly-morbid* or multicondition* or multi-condition* or ((multiple or coexist* or co-exist* or concurrent or co-occur*) adj (comorbid* or co-morbid* or "long term" or long-term or chronic or disease* or illness* or diagnos* or morbid* or condition*))).ti,ab
3. 1 or 2
4. Palliative Care/
5. Terminal Care/
6. (palliat* or terminal or "end of life" or end-of-life or dying or died or "last year" or "final year" or ((final or last) adj1 month*)).ti,ab.
7. 4 or 5 or 6
8. 3 and 7
9. (("end stage" or end-stage or "advanc*" or "complex") adj2 (multimorbid* or multi-morbid* or polymorbid* or poly-morbid* or multicondition* or multi-condition* or ((multiple or coexist* or co-exist* or concurrent or co-occur*) adj (comorbid* or co-morbid* or "long term" or long-term or chronic or disease* or illness* or diagnos* or morbid* or condition*)))).ti,ab
10. 8 or 9

For peer review only

Preferred Reporting Items for Systematic reviews and Meta-Analyses extension for Scoping Reviews (PRISMA-ScR) Checklist for this Protocol

SECTION	ITEM	PRISMA-ScR CHECKLIST ITEM	REPORTED ON PAGE #
**TITLE**			
Title	1	Identify the report as a scoping review.	1
**ABSTRACT**			
Structured summary	2	Provide a structured summary that includes (as applicable): background, objectives, eligibility criteria, sources of evidence, charting methods, results, and conclusions that relate to the review questions and objectives.	4
**INTRODUCTION**			
Rationale	3	Describe the rationale for the review in the context of what is already known. Explain why the review questions/objectives lend themselves to a scoping review approach.	5
Objectives	4	Provide an explicit statement of the questions and objectives being addressed with reference to their key elements (e.g., population or participants, concepts, and context) or other relevant key elements used to conceptualize the review questions and/or objectives.	6
**METHODS**			
Protocol and registration	5	Indicate whether a review protocol exists; state if and where it can be accessed (e.g., a Web address); and if available, provide registration information, including the registration number.	Click here to enter text.
Eligibility criteria	6	Specify characteristics of the sources of evidence used as eligibility criteria (e.g., years considered, language, and publication status), and provide a rationale.	7
Information sources*	7	Describe all information sources in the search (e.g., databases with dates of coverage and contact with authors to identify additional sources), as well as the date the most recent search was executed.	7
Search	8	Present the full electronic search strategy for at least 1 database, including any limits used, such that it could be repeated.	Supplementary information
Selection of sources of evidence†	9	State the process for selecting sources of evidence (i.e., screening and eligibility) included in the scoping review.	7
Data charting process‡	10	Describe the methods of charting data from the included sources of evidence (e.g., calibrated forms or forms that have been tested by the team before their use, and whether data charting was done independently or in duplicate) and any processes for obtaining and confirming data from investigators.	8
Data items	11	List and define all variables for which data were sought and any assumptions and simplifications made.	Click here to enter text.
Critical appraisal of individual sources of evidence§	12	If done, provide a rationale for conducting a critical appraisal of included sources of evidence; describe the methods used and how this information was used in any data synthesis (if appropriate).	Click here to enter text.
Synthesis of	13	Describe the methods of handling and summarizing	8

St. Michael's
Inspired Care.
Inspiring Science.

For peer review only - http://bmjopen.bmj.com/site/about/guidelines.xhtml

SECTION	ITEM	PRISMA-ScR CHECKLIST ITEM	REPORTED ON PAGE #
results		the data that were charted.	
**RESULTS**			
Selection of sources of evidence	14	Give numbers of sources of evidence screened, assessed for eligibility, and included in the review, with reasons for exclusions at each stage, ideally using a flow diagram.	Click here to enter text.
Characteristics of sources of evidence	15	For each source of evidence, present characteristics for which data were charted and provide the citations.	Click here to enter text.
Critical appraisal within sources of evidence	16	If done, present data on critical appraisal of included sources of evidence (see item 12).	Click here to enter text.
Results of individual sources of evidence	17	For each included source of evidence, present the relevant data that were charted that relate to the review questions and objectives.	Click here to enter text.
Synthesis of results	18	Summarize and/or present the charting results as they relate to the review questions and objectives.	Click here to enter text.
**DISCUSSION**			
Summary of evidence	19	Summarize the main results (including an overview of concepts, themes, and types of evidence available), link to the review questions and objectives, and consider the relevance to key groups.	Click here to enter text.
Limitations	20	Discuss the limitations of the scoping review process.	Click here to enter text.
Conclusions	21	Provide a general interpretation of the results with respect to the review questions and objectives, as well as potential implications and/or next steps.	Click here to enter text.
**FUNDING**			
Funding	22	Describe sources of funding for the included sources of evidence, as well as sources of funding for the scoping review. Describe the role of the funders of the scoping review.	9

JBI = Joanna Briggs Institute; PRISMA-ScR = Preferred Reporting Items for Systematic reviews and Meta-Analyses extension for Scoping Reviews.
* Where *sources of evidence* (see second footnote) are compiled from, such as bibliographic databases, social media platforms, and Web sites.
† A more inclusive/heterogeneous term used to account for the different types of evidence or data sources (e.g., quantitative and/or qualitative research, expert opinion, and policy documents) that may be eligible in a scoping review as opposed to only studies. This is not to be confused with *information sources* (see first footnote).
‡ The frameworks by Arksey and O'Malley (6) and Levac and colleagues (7) and the JBI guidance (4, 5) refer to the process of data extraction in a scoping review as data charting.
§ The process of systematically examining research evidence to assess its validity, results, and relevance before using it to inform a decision. This term is used for items 12 and 19 instead of "risk of bias" (which is more applicable to systematic reviews of interventions) to include and acknowledge the various sources of evidence that may be used in a scoping review (e.g., quantitative and/or qualitative research, expert opinion, and policy document).

From: Tricco AC, Lillie E, Zarin W, O'Brien KK, Colquhoun H, Levac D, et al. PRISMA Extension for Scoping Reviews (PRISMAScR): Checklist and Explanation. Ann Intern Med. 2018;169:467–473. doi: 10.7326/M18-0850.

For peer review only - http://bmjopen.bmj.com/site/about/guidelines.xhtml

BMJ Open

Current definitions of advanced multimorbidity: a protocol for a scoping review.

Journal:	*BMJ Open*
Manuscript ID	bmjopen-2023-076903.R1
Article Type:	Protocol
Date Submitted by the Author:	04-Oct-2023
Complete List of Authors:	Bowers, Sarah; University of St Andrews School of Medicine<br>Black, Polly; University of St Andrews School of Medicine<br>McCheyne, Lewis; NHS Fife<br>Wilson, Darcy; NHS Tayside<br>Mills, Sarah; University of St Andrews School of Medicine; NHS Fife<br>Agrawal, Utkarsh; University of Oxford Nuffield Department of Primary Care Health Sciences<br>Williams, Linda; University of Edinburgh, Usher Institute<br>Quirk, Frances; University of St Andrews School of Medicine; NHS Fife<br>Bowden, Jo; University of St Andrews School of Medicine; NHS Fife
**Primary Subject Heading**:	Palliative care
Secondary Subject Heading:	Public health
Keywords:	PALLIATIVE CARE, Decision Making, INTERNAL MEDICINE, Life Change Events, Adult palliative care < PALLIATIVE CARE, Patient-Centered Care

SCHOLARONE™
Manuscripts

Title

Current definitions of advanced multimorbidity: a protocol for a scoping review.

Dr Sarah P Bowers

Corresponding author

School of Medicine, University of St Andrews, St Andrews, UK

School of Medicine, North Haugh Street, St Andrews, KY16 9TF

01334 463599

sb461@st-andrews.ac.uk

Polly Black

School of Medicine, University of St Andrews, St Andrews, UK

pb225@st-andrews.ac.uk

Dr Lewis McCheyne

NHS Fife, Fife, UK

lewis.mccheyne@nhs.scot

Dr Darcy Wilson

NHS Tayside, Dundee, UK

darcy.wilson@nhs.scot

Dr Sarah E E Mills

School of Medicine, University of St Andrews, St Andrews, UK

NHS Fife, Fife, UK

seem1@st-andrews.ac.uk

Dr Utkarsh Agrawal

Nuffield Department of Primary Care Health Sciences, University of Oxford, Oxford, UK

utkarsh.agrawal@phc.ox.ac.uk

For peer review only

Dr Linda Williams

Edinburgh Clinical Trials Unit, Usher Institute, The University of Edinburgh, Edinburgh, UK

linda.williams@ed.ac.uk

Professor Frances Quirk

School of Medicine, University of St Andrews, St Andrews, UK

NHS Fife, Fife, UK

frances.quirk@nhs.scot

Dr Jo Bowden

School of Medicine, University of St Andrews, St Andrews, UK

NHS Fife, Fife, UK

joanna.bowden@nhs.scot

For peer review only

Word Count: 2255

Keywords:

Multimorbidity

Palliative Care

Terminal Care

Review

For peer review only

Abstract (word count = 298/300)

Introduction

People living with and dying from multimorbidity are increasing in number, and ensuring quality care for this population is one of the major challenges facing healthcare providers. People with multimorbidity often have a high burden of palliative and end of life care needs, though they do not always access specialist palliative care services. A key reason for this is that they are often not identified as being in the last stages of their life by current healthcare providers and systems.

This scoping review aims to identify and present the available evidence on how people with multimorbidity are currently included in research, policy and clinical practice.

Methods and analysis

Scoping review methodology, based on Arksey and O'Malley's framework, will be undertaken and presented using the Preferred Reporting Items for Systematic Review and Meta-Analyses extension for Scoping reviews (PRISMA-ScR). Search terms have been generated using the key themes of 'multimorbidity', 'end of life' and 'palliative care'. Peer-reviewed research will be obtained through systematic searching of Medline, EMBASE, CINAHL, Scopus and PsychINFO. Grey literature will be searched in a systematic manner. Literature containing a definition for adults with multimorbidity in a terminal phase of their illness experience will be included. After screening studies for eligibility, included studies will be described in terms of setting and characteristics as well as using inductive content analysis to highlight the commonalities in definitions.

Ethics and dissemination

Ethical approval is not required for this scoping review. The findings of the scoping review will be used internally as part of SPB's PhD thesis at the University of St Andrews through the Multimorbidity Doctoral Training Programme for Health Professionals, which is supported by the Wellcome Trust (223499/Z/21/Z) and published in an open access, peer reviewed journal for wider dissemination.

Strengths & Limitations

- This is the first scoping review to identify and map out how people with multimorbidity who are approaching the end of their life are identified in research, policy, and practice
- This protocol has been developed robustly using international guidelines and will be reported using the Preferred Reporting Items for Systematic Review and Meta-Analyses extension for Scoping Reviews (PRISMA-ScR) guidelines
- Utilising content analysis will allow for definitions from various bodies of work to be described narratively and aid future researchers, policy makers and clinicians
- A project Patient Advisory Group has been established who participated in conceptualisation of this scoping review

For peer review only

- This work is limited to English language but there is no date limit utilised

For peer review only

Introduction

Multimorbidity, defined as the presence of two or more physical or mental health conditions, is one of the main challenges facing modern healthcare systems.(1) The prevalence of multimorbidity is increasing and is particularly high in older adults, those living with socioeconomic deprivation and those with mental health disorders.(2) People living with multimorbidity are known to have poorer quality of life, increased healthcare utilisation and expenditure and shorter life expectancy.(3, 4, 5, 6) The National Institute for Care and Excellence (NICE) published guidance on managing multimorbidity with an aim to reduce treatment burden and unplanned care through providing individualised management plans and patient-centred care.(7) Whilst not focussing on how to deliver palliative and end of life care, this guidance acknowledged further research is needed to identify people with multimorbidity who have a reduced life expectancy.

People with multiple long-term conditions are inherently dealing with the prospect that their health conditions are not curable and that these may directly, or indirectly, lead to a shortening of their life expectancy.(8, 9, 10) For people with incurable single disease conditions, identification of potential unmet palliative and end of life care needs is supported by various guidelines which advocate for the proactive adoption of a palliative care approach and, where needed, early referral to palliative care services.(11, 12) Palliative care is defined by the World Health Organisation (WHO) as: "an approach that improves the quality of life of patients (adults and children) and their families who are facing problems associated with life-threatening illness."(13) Informal, often family, caregivers play a pivotal role in meeting the palliative care needs of their loved ones.(14) Palliative care can also be provided clinically by both generalists (healthcare professionals working primarily in non-Palliative Care settings) or specialists within the field of Palliative Care.(15) Involvement with Palliative Care specialists, particularly at an early stage, has been shown to have benefits on patient quality of life and in certain circumstances improve survival.(16, 17) As per the WHO definition, many people with multiple long-term conditions have been shown to have a variety of palliative care needs, and there is some evidence these are comparable to those with incurable cancer.(18, 19) Prediction modelling has also demonstrated that people with multiple long-term conditions are going to be the highest users of palliative care services by 2040.(20) Despite acknowledging the high level of need, people with multimorbidity continue to be under-referred to palliative care services, and/or referred too late.(19, 21)

Difficulty in recognising the palliative care needs of people with multimorbidity may arise from challenges in identifying when they are approaching the end of life, and pursuit of disease-modifying treatments may no longer be beneficial.(22) This so called 'advanced illness' has previously been defined in the literature as 'late-stage chronic illness when one or more conditions become serious enough that general health and function decline and treatments begin to lose their impact – a state that progresses to end of life'.(23) It is recognised that it can be a challenge to identify the end of life period for people with multimorbidity due to the unpredictable disease trajectory they often have, which may in part be due to the heterogeneity of multimorbidity itself.(24) Providing the opportunity for identification of an imminence of the end of life would allow patients with multimorbidity and their loved ones the opportunity to express preferences for care and access formalised social, health and bereavement support.(25, 26) However at present, there is no accepted definition or guidance for when in the multimorbidity illness journey a palliative care approach may be beneficial. In other words, the lack of an agreed working definition of 'advanced multimorbidity' risks people with multimorbidity who are nearing the end of life, and their caregivers, missing out on important care and support.

For peer review only

Aim & Objectives

This scoping review aims to identify and present the available evidence on how people with multimorbidity are currently included in research, policy and clinical practice. Specifically, the objectives within this scoping review are to:

- Describe how states of advanced multimorbidity are operationalised within published research and end of life care policies
- Summarise the characteristics and methodologies of the studies examining or including advanced multimorbidity
- Present the limitations and identify research gaps within this pre-established knowledge.

Methods

Scoping review methodology was selected as this particular review type has a role in conceptual mapping in healthcare, particularly in areas without a universally agreed definition.(27) This scoping review will adhere to the five-step Arksey and O'Malley framework for scoping reviews, with reference to both the updates to this from Levac et al and the Joanna Briggs Institute (JBI) guidance for conducting scoping reviews.(28, 29, 30) This scoping review was reported in accordance with the Preferred Reporting Items for Systematic Review and Meta-Analyses extension for Scoping Reviews (PRISMA-ScR).(31)

Stage 1: Identifying the Research Question

This review will particularly focus on when the goals of care of those with multimorbidity is moved from solely aiming to reverse or treat chronic conditions, and instead focuses on alleviating suffering and addressing multidimensional needs – in line with a 'palliative care approach'.(13) This is in keeping with standard accepted definitions of other life-limiting conditions and reviews on language around what encompasses an advanced illness – thus the term 'advanced multimorbidity' has been used for this review.(23, 32, 33, 34, 35) Understanding the definitions of advanced multimorbidity that are already used in research and clinical practice will allow for informed research exploring the needs of this group of patients and how they are identified for both research, clinical care and policy.

Stage 2: Identifying Relevant Studies

SPB will identify relevant studies through searches of peer-reviewed and grey literature. Search terms relating to multimorbidity, advanced illness and palliative care were developed with expertise from the University of St Andrews library service to build our search strategy, see supplementary file 1. Patients involved in health research on the impact of multimorbidity, alongside our own public advisors for this work, have denounced the term multimorbidity as having negative terms, with preferences being made for 'multiple long-term conditions', with literature often using the terms interchangeably and therefore we have integrated multiple long-term conditions into our search terms.(36) The databases Medline, EMBASE, CINAHL, Scopus and PsychINFO will be searched for published journal articles.

There will also be a search of grey literature within the United Kingdom to identify both unpublished work, indexed relevant theses and clinical guidelines and policies. Following the guidance set out by Godin et al(37), our grey literature search will comprise of a four-step approach of: searching a grey literature database – National Grey Literature Collection, supported by Health Education England(38); a contained Google search; browsing targeted websites – related to end of life care in the UK; and a

discussion amongst the clinical members of the research team (SPB, LM, DW, SM, JB) who have experience in generalist and specialist palliative care in various capacities to act as content experts and identify other items for possible inclusion in the review. Any pieces of unpublished work (e.g. conference abstracts or pre-prints) will be followed up with the authors to enquire about subsequent publication. It was deemed important to include only published research in our final texts to ensure that the studies with appropriate definitions had undergone peer-review. Given that scoping review methodology does not typically include assessment of quality, the inclusion of peer-reviewed articles allows for a step to improve the quality of included papers and the review itself.(30, 39)

The references of all included reports or articles and their subsequent citations will be searched for additional studies. There will be no date limit on our searches though we will limit to English language.

Stage 3: Study Selection

Once all searches are complete these will be combined and deduplicated using EndNote X7. Using the inclusion and exclusion criteria (Table 1), developed using the JBI Population-Concept-Context model(28), titles and abstracts will be screened by two independent reviewers (SPB and either LM or DW). We have particularly chosen to exclude studies on index conditions and comorbidity as although some argue that comorbidity and multimorbidity are interchangeable, the former is disease-focussed whereas the use of multimorbidity allows for delivery of more holistic, patient-centred care.(40) The full texts of selected citations will then be reviewed by two independent reviewers (SPB and either PB, LM or DW) to allow for approval for final study selection. Any discrepancies that may arise from each reviewer will be relayed back to a third reviewer and discussions will be held to reach consensus. Each stage of this study selection will be reported using the PRISMA-ScR Flow Chart.(31)

**Inclusion**	**Exclusion**
Population	
Adults (>18) with multimorbidity (≥2 health conditions) or their carers or healthcare providers	Studies focusing on one index condition<br>Studies focusing on comorbidity
Concept	
Definitions of multimorbidity in a terminal phase of the illness experience	Studies related solely to prognosis<br>No clear definition for advanced multimorbidity in the study/report
Context	
All settings and countries<br>Original research articles and review articles	Unpublished research<br>Not English language

Table 1 – Inclusion & Exclusion Criteria

Stage 4: Charting the Data

Data will be extracted by two independent reviewers to an Excel spreadsheet via the University of St Andrews OneDrive system to allow all researchers access – known as 'data charting' in scoping reviews.(28) Charted data will relate to study design, study population, definition of multimorbidity and reported outcomes, summarised in Table 2. This will be an iterative approach with opportunity for updating the data-charting form as informed by the literature at regular research team meetings. Given that scoping reviews aim to assess the breadth of the evidence base, formal quality assessment is not needed.(28)

For peer review only

**Study Citation Information**	Author<br>Title<br>Journal<br>Year of publication
**Study Objective**	Aim and/or objectives
**Study Methods**	Type of study
**Study Characteristics**	Country<br>Healthcare setting (if applicable)<br>Participants (number, gender, diagnoses)<br>Inclusion/exclusion criteria
**Extracted results**	Definition for advanced multimorbidity<br>Findings related to aim of study<br>Other relevant information

Table 2 – Data Charting

Stage 5: Collating, summarising and reporting the results

Once we have identified all available definitions in the literature, we will analyse the data, report the results and apply meaning to the results. We will firstly summarise the characteristics of the included work descriptively by listing the overall number of studies, distribution of the work, study populations, and countries. Using content analysis, we will explore the different definitions that are found. Content analysis allows for analysis of both quantitative and qualitative research and policies to describe phenomena to make replicable and valid inferences about the data and provide a practical guide to action. It has been successfully utilised in other scoping reviews to assimilate data on definitions.(41, 42, 43) A qualitative, inductive, content analysis approach was selected as this is preferred when there is limited knowledge about the phenomenon of choice.(44) Given there is no formally accepted standard definition of advanced multimorbidity we will undertake our content analysis inductively which involves three main phases:

- Preparation phase: wherein the unit of analysis (in this case the definition) is selected from the data using key words and phrases and a description of the context in which this was obtained
- Organisation phase: open coding is then used to collate broad categories to describe the definitions which are then organised
- Abstraction phase: this formulates a general description of the research topic through the categories above and will then be described narratively. This may include understanding how definitions vary between different disease groupings, as multimorbidity is a heterogeneous condition with variability in which combinations of conditions it is comprised of. (44)

Patient and Public Involvement

The concept and aim of this scoping review has been reviewed by our Public Advisory Group, who are members of the Fife Community Advisory Council providing patient and public involvement to the University of St Andrews. The group felt that identifying the dying phase of an individual's life was important, though also emphasised that understanding of the individual at the core of this was of most important. This lead us to utilise more holistic, patient-centred themes to our search terms (including multiple long-term conditions and the decision to focus on multimorbidity and not comorbidities). The group also feel that uncoordinated care was a challenge for people at the end of life and it is hoped this scoping review will provide operational suggestions of ways to identify people to then provide holistic, coordinated services in the future. The Public Advisory Group will contribute

For peer review only

to add their perspective to our results, particularly at the abstraction phase to ensure these are meaningful and relevant to people with lived experience.

Ethical Approval and Dissemination

This scoping review will not require ethical approval.

The findings of this scoping review will be shared through professional networks, conferences and published open access in a peer-reviewed journal.

Discussion

This scoping review aims to comprehensively analyse the currently used definitions to identify people with multimorbidity who may soon be approaching the end of life. Identifying patterns to these definitions, alongside the input of our expert focus groups, will allow for open discussion and consensus to be reached on which are the important issues to consider when identifying advanced multimorbidity. This will primarily allow for earlier advanced care planning and opportunities for specialist care for patients with multimorbidity as well as ensuring more consistent research is undertaken and impact on national and international policy development.

Acknowledgements

We are extremely grateful to Vicki Cormie, Senior Librarian at University of St Andrews, for her support and guidance in the development of the search strategy and to our public advisory team who have provided their expert lived opinion to the development of this scoping review. Particular thanks also to Rosie Dunn for her guidance on using content analysis within scoping reviews.

Contributorship Statement

SPB developed the concept of the scoping review, research questions, public advisory group discussions and drafted the manuscript. Input to conceptualisation was also given by SEEM, UA, LW, FQ and JB. SEEM and UA assisted with development of the search strategy. SPB, LM, DW, SEEM and JB provided content guidance through their clinical expertise. SPB drafted the manuscript with PB, LM, DW, FQ and JB critically reviewing drafts of this. All authors approved the final submitted manuscript and agree to be accountable for all aspects of this protocol.

Competing Interests

None declared.

Funding Statement

For peer review only

This review will contribute to the primary author's (SPB) PhD Thesis. SPB is a fellow on the Multimorbidity Doctoral Training Programme for Health Professionals, which is supported by the Wellcome Trust (223499/Z/21/Z), and supervised by SEEM, UA, LW, JB and FQ. PB is also a fellow on the Multimorbidity Doctoral Training Programme for Health Professionals, which is supported by the Wellcome Trust (223499/Z/21/Z). As this research was funded in whole, or in part, by the Wellcome Trust (223499/Z/21/Z), for the purpose of open access, the author has applied a CC BY public copyright licence to any Author Accepted Manuscript version arising from this submission.

For peer review only

References

1. Whitty CJM, MacEwen C, Goddard A, Alderson D, Marshall M, Calderwood C, et al. Rising to the challenge of multimorbidity. Bmj. 2020;368:l6964.
2. Barnett K, Mercer SW, Norbury M, Watt G, Wyke S, Guthrie B. Epidemiology of multimorbidity and implications for health care, research, and medical education: a cross-sectional study. Lancet. 2012;380(9836):37-43.
3. Lawson KD, Mercer SW, Wyke S, Grieve E, Guthrie B, Watt GC, et al. Double trouble: the impact of multimorbidity and deprivation on preference-weighted health related quality of life a cross sectional analysis of the Scottish Health Survey. Int J Equity Health. 2013;12:67.
4. Soley-Bori M, Ashworth M, Bisquera A, Dodhia H, Lynch R, Wang Y, et al. Impact of multimorbidity on healthcare costs and utilisation: a systematic review of the UK literature. Br J Gen Pract. 2021;71(702):e39-e46.
5. Johnston MC, Black C, Mercer SW, Prescott GJ, Crilly MA. Prevalence of secondary care multimorbidity in mid-life and its association with premature mortality in a large longitudinal cohort study. BMJ Open. 2020;10(5):e033622.
6. He K, Zhang W, Hu X, Zhao H, Guo B, Shi Z, et al. Relationship between multimorbidity, disease cluster and all-cause mortality among older adults: a retrospective cohort analysis. BMC Public Health. 2021;21(1):1080.
7. NICE. Multimorbidity: clinical assessment and management 2016 [Available from: https://www.nice.org.uk/guidance/ng56.
8. Yardley S, Cottrell E, Rees E, Protheroe J. Modelling successful primary care for multimorbidity: a realist synthesis of successes and failures in concurrent learning and healthcare delivery. BMC Fam Pract. 2015;16:23.
9. Navickas R, Petric VK, Feigl AB, Seychell M. Multimorbidity: What do we know? What should we do? J Comorb. 2016;6(1):4-11.
10. Brijoux T, Woopen C, Zank S. Multimorbidity in old age and its impact on life results. Z Gerontol Geriatr. 2021;54(Suppl 2):108-13.
11. Ferrell BR, Temel JS, Temin S, Alesi ER, Balboni TA, Basch EM, et al. Integration of Palliative Care Into Standard Oncology Care: American Society of Clinical Oncology Clinical Practice Guideline Update. J Clin Oncol. 2017;35(1):96-112.
12. Jordan K, Aapro M, Kaasa S, Ripamonti CI, Scotté F, Strasser F, et al. European Society for Medical Oncology (ESMO) position paper on supportive and palliative care. Ann Oncol. 2018;29(1):36-43.
13. World Health Organisation. Palliative care 2020 [Available from: https://www.who.int/news-room/fact-sheets/detail/palliative-care.
14. Ateş G, Ebenau AF, Busa C, Csikos Á, Hasselaar J, Jaspers B, et al. "Never at ease" - family carers within integrated palliative care: a multinational, mixed method study. BMC Palliat Care. 2018;17(1):39.
15. NICE. Palliative care - general issues: What is palliative care? 2022 [Available from: https://cks.nice.org.uk/topics/palliative-care-general-issues/background-information/definition/.
16. Gaertner J, Siemens W, Meerpohl JJ, Antes G, Meffert C, Xander C, et al. Effect of specialist palliative care services on quality of life in adults with advanced incurable illness in hospital, hospice, or community settings: systematic review and meta-analysis. Bmj. 2017;357:j2925.
17. Temel JS, Greer JA, Muzikansky A, Gallagher ER, Admane S, Jackson VA, et al. Early palliative care for patients with metastatic non-small-cell lung cancer. N Engl J Med. 2010;363(8):733-42.
18. Nicholson C, Davies JM, George R, Smith B, Pace V, Harris L, et al. What are the main palliative care symptoms and concerns of older people with multimorbidity?-a comparative cross-sectional study using routinely collected Phase of Illness, Australia-modified Karnofsky Performance Status and Integrated Palliative Care Outcome Scale data. Ann. 2018;7(Suppl 3):S164-S75.

For peer review only

19. Llop-Medina L, Fu Y, Garces-Ferrer J, Donate-Martinez A. Palliative Care in Older People with Multimorbidities: A Scoping Review on the Palliative Care Needs of Patients, Carers, and Health Professionals. Int J Environ Res Public Health. 2022;19(6):08.
20. Finucane AM, Bone AE, Etkind S, Carr D, Meade R, Munoz-Arroyo R, et al. How many people will need palliative care in Scotland by 2040? A mixed-method study of projected palliative care need and recommendations for service delivery. BMJ Open. 2021;11(2):e041317.
21. Carduff E, Johnston S, Winstanley C, Morrish J, Murray SA, Spiller J, et al. What does 'complex' mean in palliative care? Triangulating qualitative findings from 3 settings. BMC Palliat Care. 2018;17(1):12.
22. Tobin J, Rogers A, Winterburn I, Tullie S, Kalyanasundaram A, Kuhn I, et al. Hospice care access inequalities: a systematic review and narrative synthesis. BMJ Supportive & Palliative Care. 2022;12(2):142-51.
23. Stuart B, Mahler E, Koomson P. A Large-Scale Advanced Illness Intervention Informs Medicare's New Serious Illness Payment Model. Health Aff (Millwood). 2019;38(6):950-6.
24. Teraoka E, Kunisawa S, Imanaka Y. Trajectories of end-of-life medical and long-term care expenditures for older adults in Japan: retrospective longitudinal study using a large-scale linked database of medical and long-term care claims. BMC geriatr. 2021;21(1):403.
25. NICE. Quality standard [QS13]. End of life care for adults: National Institute for Health and Care Excellence; [updated 02/09/2021. Available from: https://www.nice.org.uk/guidance/qs13#:~:text=This%20quality%20standard%20covers%20care,with%20life%2Dthreatening%20acute%20conditions.
26. Kennedy C, Brooks-Young P, Brunton Gray C, Larkin P, Connolly M, Wilde-Larsson B, et al. Diagnosing dying: an integrative literature review. BMJ Supportive & Palliative Care. 2014;4(3):263-70.
27. Anderson S, Allen P, Peckham S, Goodwin N. Asking the right questions: scoping studies in the commissioning of research on the organisation and delivery of health services. Health Res Policy Syst. 2008;6:7.
28. Peters MD, Godfrey CM, Khalil H, McInerney P, Parker D, Soares CB. Guidance for conducting systematic scoping reviews. Int J Evid Based Healthc. 2015;13(3):141-6.
29. Arksey H, O'Malley L. Scoping studies: towards a methodological framework. International Journal of Social Research Methodology. 2005;8(1):19-32.
30. Levac D, Colquhoun H, O'Brien KK. Scoping studies: advancing the methodology. Implement Sci. 2010;5:69.
31. Tricco AC, Lillie E, Zarin W, O'Brien KK, Colquhoun H, Levac D, et al. PRISMA Extension for Scoping Reviews (PRISMA-ScR): Checklist and Explanation. Ann Intern Med. 2018;169(7):467-73.
32. Hudson RB, Lindberg BW. Advanced Illness Care: Issues and Options. Public Policy & Aging Report. 2014;24(3):79-80.
33. Bruera E, Yennurajalingam S. Palliative care in advanced cancer patients: how and when? Oncologist. 2012;17(2):267-73.
34. Traue DC, Ross JR. Palliative care in non-malignant diseases. J R Soc Med. 2005;98(11):503-6.
35. Hui D, Nooruddin Z, Didwaniya N, Dev R, De La Cruz M, Kim SH, et al. Concepts and definitions for "actively dying," "end of life," "terminally ill," "terminal care," and "transition of care": a systematic review. J Pain Symptom Manage. 2014;47(1):77-89.
36. Chew-Graham C, O'Toole L, Taylor J, Salisbury C. 'Multimorbidity': an acceptable term for patients or time for a rebrand? Br J Gen Pract. 2019;69(685):372-3.
37. Godin K, Stapleton J, Kirkpatrick SI, Hanning RM, Leatherdale ST. Applying systematic review search methods to the grey literature: a case study examining guidelines for school-based breakfast programs in Canada. Syst. 2015;4(1):138.
38. Health Education England. National Grey Literature Collection 2023 [Available from: https://allcatsrgrey.org.uk/wp/.

For peer review only

39. Kelly J, Sadeghieh T, Adeli K. Peer Review in Scientific Publications: Benefits, Critiques, & A Survival Guide. Ejifcc. 2014;25(3):227-43.
40. Harrison C, Fortin M, van den Akker M, Mair F, Calderon-Larranaga A, Boland F, et al. Comorbidity versus multimorbidity: Why it matters. J Multimorb Comorb. 2021;11:2633556521993993.
41. Dunn R, Clayton E, Wolverson E, Hilton A. Conceptualising comorbidity and multimorbidity in dementia: A scoping review and syndemic framework. J Multimorb Comorb. 2022;12:26335565221128432.
42. Kwok E, Bootsma J, Cahill P, Rosenbaum P. A scoping review of qualitative studies on parents' perspectives on speech, language, and communication interventions. Disability and Rehabilitation. 2022;44(25):8084-93.
43. Churruca K, Pomare C, Ellis LA, Long JC, Braithwaite J. The influence of complexity: a bibliometric analysis of complexity science in healthcare. BMJ Open. 2019;9(3):e027308.
44. Elo S, Kyngäs H. The qualitative content analysis process. J Adv Nurs. 2008;62(1):107-15.

For peer review only

Medline and Embase

1. Multimorbidity/
2. (multimorbid* or multi-morbid* or polymorbid* or poly-morbid* or multicondition* or multi-condition* or ((multiple or coexist* or co-exist* or concurrent or co-occur*) adj (comorbid* or co-morbid* or "long term" or long-term or chronic or disease* or illness* or diagnos* or morbid* or condition*))).ti,ab
3. 1 or 2
4. Palliative Care/
5. Terminal Care/
6. (palliat* or terminal or "end of life" or end-of-life or dying or died or "last year" or "final year" or ((final or last) adj1 month*)).ti,ab.
7. 4 or 5 or 6
8. 3 and 7
9. (("end stage" or end-stage or "advanc*" or "complex") adj2 (multimorbid* or multi-morbid* or polymorbid* or poly-morbid* or multicondition* or multi-condition* or ((multiple or coexist* or co-exist* or concurrent or co-occur*) adj (comorbid* or co-morbid* or "long term" or long-term or chronic or disease* or illness* or diagnos* or morbid* or condition*)))).ti,ab
10. 8 or 9

This search strategy has been chosen as described in our Methods section to capture key papers around themes of 'multimorbidity', 'end of life' and 'palliative care'.

Steps 1-3 capture multimorbidity

Steps 4-7 capture end of life care and palliative care

Step 8 then combines the above phrases to focus these above points on when multimorbidity is described at the end of life or in a palliative care context

Step 9 captures papers which specifically mention advanced (or other synonymous phrases) multimorbidity

Steps 8 and 10 is then our final included papers in the search strategy

For peer review only

Preferred Reporting Items for Systematic reviews and Meta-Analyses extension for Scoping Reviews (PRISMA-ScR) Checklist for this Protocol

SECTION	ITEM	PRISMA-ScR CHECKLIST ITEM	REPORTED ON PAGE #
**TITLE**			
Title	1	Identify the report as a scoping review.	1
**ABSTRACT**			
Structured summary	2	Provide a structured summary that includes (as applicable): background, objectives, eligibility criteria, sources of evidence, charting methods, results, and conclusions that relate to the review questions and objectives.	4
**INTRODUCTION**			
Rationale	3	Describe the rationale for the review in the context of what is already known. Explain why the review questions/objectives lend themselves to a scoping review approach.	5
Objectives	4	Provide an explicit statement of the questions and objectives being addressed with reference to their key elements (e.g., population or participants, concepts, and context) or other relevant key elements used to conceptualize the review questions and/or objectives.	6
**METHODS**			
Protocol and registration	5	Indicate whether a review protocol exists; state if and where it can be accessed (e.g., a Web address); and if available, provide registration information, including the registration number.	Click here to enter text.
Eligibility criteria	6	Specify characteristics of the sources of evidence used as eligibility criteria (e.g., years considered, language, and publication status), and provide a rationale.	7
Information sources*	7	Describe all information sources in the search (e.g., databases with dates of coverage and contact with authors to identify additional sources), as well as the date the most recent search was executed.	7
Search	8	Present the full electronic search strategy for at least 1 database, including any limits used, such that it could be repeated.	Supplementary information
Selection of sources of evidence†	9	State the process for selecting sources of evidence (i.e., screening and eligibility) included in the scoping review.	7
Data charting process‡	10	Describe the methods of charting data from the included sources of evidence (e.g., calibrated forms or forms that have been tested by the team before their use, and whether data charting was done independently or in duplicate) and any processes for obtaining and confirming data from investigators.	8
Data items	11	List and define all variables for which data were sought and any assumptions and simplifications made.	Click here to enter text.
Critical appraisal of individual sources of evidence§	12	If done, provide a rationale for conducting a critical appraisal of included sources of evidence; describe the methods used and how this information was used in any data synthesis (if appropriate).	Click here to enter text.
Synthesis of	13	Describe the methods of handling and summarizing	8

For peer review only

SECTION	ITEM	PRISMA-ScR CHECKLIST ITEM	REPORTED ON PAGE #
results		the data that were charted.	
**RESULTS**			
Selection of sources of evidence	14	Give numbers of sources of evidence screened, assessed for eligibility, and included in the review, with reasons for exclusions at each stage, ideally using a flow diagram.	Click here to enter text.
Characteristics of sources of evidence	15	For each source of evidence, present characteristics for which data were charted and provide the citations.	Click here to enter text.
Critical appraisal within sources of evidence	16	If done, present data on critical appraisal of included sources of evidence (see item 12).	Click here to enter text.
Results of individual sources of evidence	17	For each included source of evidence, present the relevant data that were charted that relate to the review questions and objectives.	Click here to enter text.
Synthesis of results	18	Summarize and/or present the charting results as they relate to the review questions and objectives.	Click here to enter text.
**DISCUSSION**			
Summary of evidence	19	Summarize the main results (including an overview of concepts, themes, and types of evidence available), link to the review questions and objectives, and consider the relevance to key groups.	Click here to enter text.
Limitations	20	Discuss the limitations of the scoping review process.	Click here to enter text.
Conclusions	21	Provide a general interpretation of the results with respect to the review questions and objectives, as well as potential implications and/or next steps.	Click here to enter text.
**FUNDING**			
Funding	22	Describe sources of funding for the included sources of evidence, as well as sources of funding for the scoping review. Describe the role of the funders of the scoping review.	9

JBI = Joanna Briggs Institute; PRISMA-ScR = Preferred Reporting Items for Systematic reviews and Meta-Analyses extension for Scoping Reviews.
* Where *sources of evidence* (see second footnote) are compiled from, such as bibliographic databases, social media platforms, and Web sites.
† A more inclusive/heterogeneous term used to account for the different types of evidence or data sources (e.g., quantitative and/or qualitative research, expert opinion, and policy documents) that may be eligible in a scoping review as opposed to only studies. This is not to be confused with *information sources* (see first footnote).
‡ The frameworks by Arksey and O'Malley (6) and Levac and colleagues (7) and the JBI guidance (4, 5) refer to the process of data extraction in a scoping review as data charting.
§ The process of systematically examining research evidence to assess its validity, results, and relevance before using it to inform a decision. This term is used for items 12 and 19 instead of "risk of bias" (which is more applicable to systematic reviews of interventions) to include and acknowledge the various sources of evidence that may be used in a scoping review (e.g., quantitative and/or qualitative research, expert opinion, and policy document).

From: Tricco AC, Lillie E, Zarin W, O'Brien KK, Colquhoun H, Levac D, et al. PRISMA Extension for Scoping Reviews (PRISMAScR): Checklist and Explanation. Ann Intern Med. 2018;169:467–473. doi: 10.7326/M18-0850.

For peer review only - http://bmjopen.bmj.com/site/about/guidelines.xhtml
